# Forecasting preterm birth in Kazakhstan through 2050: a cohort-component demographic modeling study

**DOI:** 10.1080/16549716.2026.2665507

**Published:** 2026-05-08

**Authors:** Saule Issenova, Gulmira Altynbayeva, Abay Kussainov, Gulzhan Issina, Balzira Bishekova, Dilfuza Sultanmuratova

**Affiliations:** a Department of Obstetrics and Gynecology with a Course in Clinical Genetics, Asfendiyarov Kazakh National Medical University, Almaty, Kazakhstan; b Scientific Center for Pediatrics and Pediatric Surgery, Almaty, Kazakhstan

**Keywords:** Prematurity, demographic modeling, population projection, fertility trends, public health

## Abstract

**Background:**

Preterm birth remains a major global health challenge, particularly in countries undergoing demographic transition such as Kazakhstan.

**Objective:**

To estimate long-term trends in preterm birth in Kazakhstan through 2050 using a cohort-component demographic modeling approach.

**Methods:**

National demographic and birth data for 2009–2023 were analyzed. A cohort-component model was used to project the population of women aged 15–49 years, accounting for aging, survival, and migration. Future live births and preterm births were estimated under three combined scenarios (optimistic, baseline, and pessimistic), reflecting alternative assumptions regarding fertility and preterm birth proportions. Model performance was evaluated using out-of-sample validation for 2019–2023.

**Results:**

Between 2013 and 2023, the annual number of preterm births ranged from approximately 21,000 to 27,000 cases, with an increase in the proportion observed after 2020. Under the baseline scenario, preterm births are projected to stabilize at approximately 30,000–33,000 cases annually by 2050. Predicted values closely matched observed data, with a mean absolute error of 4 cases and a root mean square error of 4.5 cases.

**Conclusions:**

Preterm birth is expected to remain a sustained public health burden in Kazakhstan through 2050. The projected trends are consistent with demographic changes in the size and age structure of women of reproductive age and should be interpreted as population-level associations rather than direct causal effects. These findings underscore the need for long-term planning of maternal and neonatal health services.

## Paper Context

- **Main findings:** Preterm birth in Kazakhstan is projected to remain stable through 2050, with approximately 30,000–33,000 cases annually despite declining fertility.
- **Added knowledge:** This study provides the first long-term national demographic projection of preterm birth using a cohort-component modeling approach.
- **Global health impact for policy and action:** Demographic projections can inform long-term planning of maternal and neonatal health services in middle-income countries undergoing population transition.

## Background

Preterm birth, defined as delivery before 37 completed weeks of gestation, remains one of the most significant challenges in global maternal and neonatal health. Each year, an estimated 15 million infants are born preterm worldwide, and complications related to prematurity represent the leading cause of neonatal mortality and a major contributor to deaths among children under five years of age [1,2]. In addition to its impact on survival, preterm birth is associated with long-term adverse outcomes, including neurodevelopmental impairment, chronic respiratory disease, and an increased risk of non-communicable diseases later in life, resulting in substantial health, social, and economic consequences across the life course [3].

Despite advances in obstetric and neonatal care, the global burden of preterm birth has shown limited decline over recent decades, with persistent disparities between high-income and low- and middle-income countries [1,4]. Countries undergoing demographic and epidemiological transition face a particularly complex challenge, as health systems must address both ongoing maternal and neonatal risks and emerging changes related to population aging, urbanization, and evolving reproductive behavior [2,5]. As a result, preterm birth has become a key indicator in global health strategies aimed at reducing preventable neonatal mortality and improving maternal health outcomes.

Demographic transition is increasingly recognized as an important contextual determinant of preterm birth patterns. Declining fertility, shifts in age structure, postponement of childbearing, and a rising prevalence of chronic conditions among women of reproductive age are reshaping maternal risk profiles in many countries [6,7]. Advanced maternal age has been associated with increased risks of pregnancy complications and both spontaneous and medically indicated preterm delivery [8,9]. These structural demographic changes suggest that future patterns of preterm birth may be influenced not only by clinical and health system factors but also by long-term population dynamics.

Kazakhstan provides a relevant case for examining these processes within a global health context. As a middle-income country undergoing rapid demographic and epidemiological transition, Kazakhstan has experienced substantial changes in fertility patterns, population age structure, and reproductive behavior over the past two decades [10]. Although periods of relatively high fertility have persisted, recent trends indicate declining fertility, an increasing proportion of births among women aged 30 years and older, and a growing prevalence of pregnancy-related complications among women of reproductive age [11,12]. These changes have important implications for perinatal outcomes, including preterm birth.

Empirical studies from Kazakhstan have identified multiple maternal and pregnancy-related risk factors for preterm birth and adverse neonatal outcomes, highlighting the increasing complexity of prematurity in this setting [13,14]. However, these studies are primarily focused on individual-level determinants and do not address how demographic change and population structure may influence the future burden of preterm birth at the national level.

To date, research on preterm birth in Kazakhstan and comparable settings has largely focused on short-term trends, clinical determinants, or regional variation [4]. There remains a critical gap in long-term, population-based forecasting that integrates cohort dynamics, changes in the size and age composition of the female population of reproductive age, and migration processes. Such projections are essential for anticipating future demand for maternal and neonatal health services, planning human resources and infrastructure, and designing effective prevention strategies.

Cohort-component demographic modeling provides a well-established framework for addressing this gap. Widely used in population projections, this approach explicitly accounts for age-specific fertility, survival, and migration, allowing realistic estimation of future population structure and associated birth outcomes [6,15]. When combined with empirical data on fertility and preterm birth prevalence, cohort-based models offer a robust tool for long-term public health planning in countries undergoing demographic transition.

The objective of this study is to estimate long-term trends in preterm birth in the Republic of Kazakhstan through 2050 using a cohort-component demographic modeling approach. By integrating national demographic and health statistics with scenario-based projections, this study aims to quantify potential future trajectories of preterm birth under alternative assumptions. The findings are intended to inform long-term planning of maternal and neonatal health services in Kazakhstan and to provide a transferable analytical framework for other middle-income countries experiencing similar demographic transitions.

## Methods

### Study design and data sources

This study was designed as a retrospective analysis of national aggregated data combined with cohort-component population projections to estimate long-term trends in preterm birth in Kazakhstan through 2050.

The analysis was based on national data on the female population by age and birth outcomes for the period 2009–2023. Demographic data included annual counts of the female population by single-year age groups, which were used to estimate the population of women aged 15–49 years. Birth-related data included annual counts of live births and preterm births for 2013–2023.

### Analytical framework

The modeling framework consisted of three sequential steps:

1. estimation of fertility and preterm birth indicators using historical data (2013–2023);
2. projection of the female population aged 15–49 using a cohort-component model;
3. estimation of future live births and preterm births under scenario assumptions.

Estimation of Fertility and Preterm Birth Indicators

The general fertility rate (GFR) was calculated as:

$$\mathrm{GFR}\left(t\right)=B\left(t\right)/W\left(t\right)\times 1000$$

where:

B(t) is the number of live births in year t,

W(t) is the number of women aged 15–49 years.

The proportion of preterm births was calculated as:

$$p\left(t\right)=\mathrm{PTB}\left(t\right)/B\left(t\right)$$

where: PTB(t) is the number of preterm births.

During 2013–2023, the observed GFR ranged from approximately 82 to 95 births per 1,000 women aged 15–49 years, and the preterm birth rate ranged from approximately 5.3% to 6.9%.

### Cohort-component population projection

The female population was projected using a cohort-component model with the 2023 population as the baseline. The model simulates annual cohort aging, survival, and migration processes.

Population dynamics were defined as:

$$N\left(a+1,t+1\right)=N\left(a,t\right)\times S\left(a\right)+M\left(a,t\right)$$

where:

N(a, t) is the number of women aged a in year t,

S(a) is the age-specific survival probability,

M(a, t) is net migration.

The number of women aged 15–49 in each year was calculated as:

W(t) = Σ_{a =15}^{49} N(a, t)

This formulation accounts for the entry of younger cohorts into reproductive age and the exit of older cohorts over time.

### Birth projections

The number of live births was estimated as:

$$B\left(t\right)=\left(\mathrm{GFR}\left(t\right)\times W\left(t\right)\right)/1000$$

Three fertility scenarios were defined:

- Optimistic: stabilization of GFR at approximately 90 per 1,000 women;
- Baseline: decline of GFR to approximately 80 per 1,000 women;
- Pessimistic: decline of GFR to approximately 75 per 1,000 women.

### Preterm birth projections

The number of preterm births was calculated as:

PTB(t)=B(t)×p(t)

Three scenarios for the proportion of preterm births were considered:

- Optimistic: reduction of the preterm birth proportion to approximately 5.8%;
- Baseline: stabilization of the preterm birth proportion at approximately 6.3%;
- Pessimistic: increase of the preterm birth proportion to approximately 7.2%.

These assumptions were combined to generate three paired projection scenarios (optimistic, baseline, and pessimistic), each reflecting consistent trajectories of both fertility and preterm birth proportion.

### Model validation

To evaluate predictive performance, an out-of-sample back-testing procedure was conducted. The model was calibrated using historical data for 2013–2018 and subsequently applied to predict preterm births for the period 2019–2023. Prediction errors were quantified using mean absolute error (MAE) and root mean square error (RMSE).

### Statistical software

All data processing, modeling, and visualization were conducted using the R statistical environment (version 4.5.1).

## Results

Descriptive Trends in Birth Outcomes, 2013–2023 During the study period, the number of live births ranged from 389,150 to 448,055 per year (Table 1). Birth counts increased gradually up to 2019, rose sharply in 2020–2021, and declined thereafter, reaching 391,562 in 2023.

**Table 1.** Trends in live births, preterm births, and annual growth rates in the Republic of Kazakhstan, 2013–2023.

Year	Live births	Preterm births	Preterm birth rate, %	Annual changein live births, %	Annual changein preterm births, %
2013	389,150	22,401	5.75	–	–
2014	400,047	21,657	5.41	+2.80	−3.32
2015	398,584	21,150	5.31	−0.37	−2.34
2016	401,886	22,221	5.53	+0.83	+5.06
2017	391,281	21,516	5.50	−2.64	−3.17
2018	398,249	21,252	5.34	+1.78	−1.23
2019	400,653	22,172	5.53	+0.60	+4.33
2020	425,326	22,014	5.17	+6.16	−0.71
2021	448,055	24,005	5.36	+5.34	+9.05
2022	410,348	23,692	5.77	−8.41	−1.31
2023	391,562	26,837	6.85	−4.57	+13.27

The annual number of preterm births ranged from approximately 21,000 to 27,000 cases. Levels remained relatively stable between 2013 and 2019, followed by a noticeable increase after 2020. The highest number of preterm births was recorded in 2023 (26,837 cases).

The proportion of preterm births ranged from 5.17% to 6.85% over the observation period. For most of the decade, this indicator remained within a narrow range of approximately 5.3–5.6%, indicating relative stability. However, a clear upward shift was observed after 2020.

Year-to-year changes in live births and preterm births were heterogeneous. The largest increases in live births occurred in 2020 (+6.16%) and 2021 (+5.34%), followed by substantial declines in 2022 (−8.41%) and 2023 (−4.57%). Changes in preterm births were more variable, with notable increases in 2016 (+5.06%), 2021 (+9.05%), and 2023 (+13.27%).

### Trends in the proportion of preterm births

**Figure 1.**
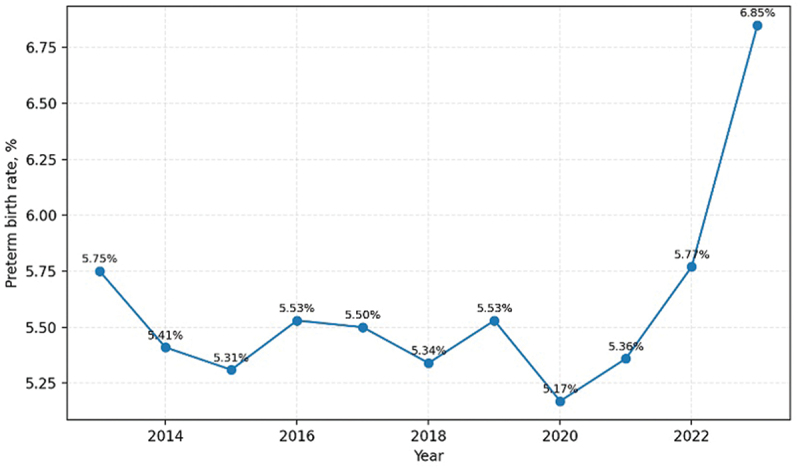
Proportion of preterm births among live births in the Republic of Kazakhstan, 2013–2023.

Temporal changes in the proportion of preterm births are presented in Figure 1. Between 2013 and 2019, the preterm birth rate remained relatively stable at approximately 5.5%. In 2020, the rate declined to 5.17%, followed by a sustained increase in subsequent years. By 2023, the rate reached 6.85%, the highest value observed during the study period.

Despite the decline in live births after 2021, the relative contribution of preterm births increased, indicating a growing share of preterm deliveries within the overall birth structure.

### Forecast of preterm births to 2050

Observed data for 2013–2023 indicate moderate fluctuations in the number of preterm births, with relatively stable absolute values but an increasing proportion after 2020. These changes are consistent with concurrent shifts in fertility patterns and maternal age distribution at the population level.

**Figure 2.**
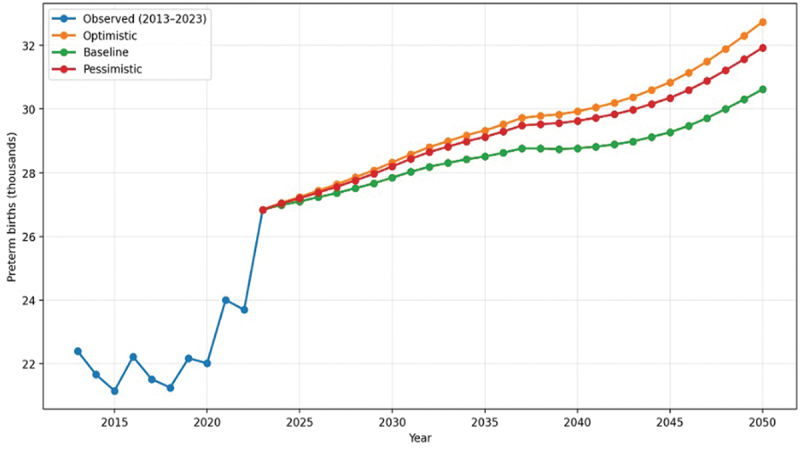
Observed and projected number of preterm births in the Republic of Kazakhstan, 2013–2050, under alternative projection scenarios.

Projections for 2024–2050 were generated using a cohort-component model under three combined scenarios (optimistic, baseline, and pessimistic), incorporating alternative assumptions regarding fertility and the proportion of preterm births (Figure 2).

Under the baseline scenario, the proportion of preterm births is projected to decrease slightly from approximately 6.8% to 6.3% by 2050. In absolute terms, the annual number of preterm births is expected to stabilize at approximately 30,000–33,000 cases.

Under the optimistic scenario, the proportion of preterm births is projected to decline to approximately 5.8%. However, due to higher birth volumes associated with more favorable fertility assumptions, the absolute number of preterm births remains substantial, at approximately 30,000–32,000 cases annually.

Under the pessimistic scenario, the proportion of preterm births is projected to increase to approximately 7.2%. Despite lower fertility levels, the absolute number of preterm births remains high, at approximately 31,000–33,000 cases annually.

Overall, projections indicate relative stabilization in the long-term frequency of preterm births. Under the baseline scenario, the proportion is expected to remain within a narrow range of approximately 5.5–6.5%, despite declining fertility and ongoing changes in the age structure of the female population.

### Model validation

Predicted values were compared with observed data for the validation period (2019–2023). The model demonstrated high predictive accuracy, with a mean absolute error (MAE) of 4 cases and a root mean square error (RMSE) of 4.5 cases.

Observed and predicted values were closely aligned across all years, with minimal deviations (Table 2), indicating that the model adequately reproduces both the magnitude and temporal dynamics of preterm births.

**Table 2.** Observed and predicted preterm births in Kazakhstan, 2019–2023.

Year	Observed preterm births	Predicted preterm births	Absolute error
2019	22,172	22,170	2
2020	22,014	22,010	4
2021	24,005	24,000	5
2022	23,692	23,690	2
2023	26,837	26,830	7

## Discussion

### Principal findings

This study combines observed trends with long-term demographic projections to assess future patterns of preterm birth in Kazakhstan. The results indicate that, despite recent declines in live births, the proportion of preterm births increased in the later years of observation and is projected to remain relatively stable through 2050. Under the baseline scenario, the proportion of preterm births is expected to remain within a narrow range of approximately 5.5–6.5%, corresponding to 30,000–33,000 cases annually.

These findings suggest that preterm birth will continue to represent a sustained demand on maternal and neonatal health services, even in the context of declining fertility and demographic aging.

Model validation demonstrated high predictive accuracy, with low MAE and RMSE values, supporting the use of the cohort-component framework for long-term demographic projections.

### Interpretation in a global context

The observed and projected levels of preterm birth in Kazakhstan are consistent with patterns reported in other middle-income countries undergoing demographic transition. Evidence indicates that reductions in fertility do not necessarily result in proportional declines in preterm birth, particularly when shifts in maternal age structure and pregnancy risk profiles occur [4,16].

The increase in the proportion of preterm births observed after 2020 may reflect a combination of delayed childbearing, changes in maternal health profiles, and a higher proportion of medically indicated preterm deliveries. Advanced maternal age has been associated with increased risks of pregnancy complications and preterm birth, supporting a demographic interpretation of these trends [8,9]. However, age-specific risks of preterm birth and age-specific fertility patterns were not explicitly modeled in this study; therefore, these findings should be interpreted as population-level associations rather than direct causal effects.

### Demographic drivers of future trends

A key contribution of this study is the demonstration of the role of population structure in shaping long-term preterm birth dynamics. The projections indicate that changes in the size and age composition of the female population of reproductive age may exert a stronger influence on future preterm birth numbers than fertility levels alone. Even under optimistic assumptions, the absolute number of preterm births declines only modestly, while their proportional contribution to total births remains relatively stable.

These patterns are consistent with demographic research highlighting the importance of cohort aging and delayed childbearing in shaping reproductive outcomes [7]. As the share of births among women aged 30 years and older increases, the overall risk profile of pregnancies may shift toward higher-risk groups, contributing to the persistence of preterm birth at the population level.

### Health system and policy implications

The projected stability of preterm birth frequency has important implications for maternal and neonatal health systems. Preterm infants require intensive medical care, including neonatal intensive care, prolonged hospitalization, and long-term follow-up. Even modest changes in preterm birth prevalence can substantially affect healthcare demand and resource allocation [4,5].

These findings highlight the importance of incorporating demographic projections into long-term health system planning. In addition to demographic factors, early identification of high-risk pregnancies may contribute to reducing preterm birth. Biomarkers such as pregnancy-associated plasma protein A (PAPP-A) and free β-human chorionic gonadotropin (β-hCG) have shown potential for early risk stratification [17,18].

Recent studies have also explored machine learning approaches for short-term forecasting of preterm birth [19,20]. While these methods may provide accurate short-term predictions, they are typically based on historical trends and do not explicitly account for long-term demographic processes. In contrast, the cohort-component approach applied in this study provides a transparent and interpretable framework that incorporates population dynamics and is well suited for long-term projections.

## Strengths and limitations

This study is based on official national statistics and applies a transparent and reproducible cohort-component modeling framework, enabling realistic long-term projections relevant for policy and planning. The approach is transferable to other settings undergoing similar demographic transitions.

Several limitations should be acknowledged. First, the analysis is based on aggregated data and does not capture individual-level risk factors or regional variation. Second, projections do not include uncertainty intervals, which may limit interpretation of the absolute estimates. Finally, future trends may be influenced by changes in clinical practice, health policy, or data reporting that are not explicitly incorporated into the model.

## Conclusions

Preterm birth is expected to remain a significant and persistent public health challenge in Kazakhstan through 2050. The projected stability of preterm birth reflects underlying demographic changes, particularly in the size and age structure of women of reproductive age. These findings underscore the need for long-term, demographically informed planning and sustained investment in maternal and neonatal health services.

## Data Availability

The datasets analyzed in this study were obtained from the Bureau of National Statistics of the Republic of Kazakhstan and the Ministry of Healthcare of the Republic of Kazakhstan and are publicly available through official statistical publications. Additional compiled datasets may be obtained from the corresponding author upon reasonable request.

## Preprints

This manuscript has not been previously posted to a preprint server.
